# Supplementary material for: Phosphate Removal Mechanisms in Aqueous Solutions by Three Different Fe-Modified Biochars
Source: Int J Environ Res Public Health. 2022 Dec 25;20(1):326. doi: 10.3390/ijerph20010326 (PMC9820018; doi:10.3390/ijerph20010326)
Supplement: Supplementary file 1 [file ijerph-20-00326-s001.zip › ijerph-2108302-supplementary.pdf]

Supplementary Materials

Phosphate Removal Mechanisms in Aqueous Solutions by Three Different Fe-Modified Biochars

Yiyin Qin [1,2], Xinyi Wu [1,2], Qiqi Huang [1,2], Jingzi Beiyuan [1,3,*], Jin Wang [4], Juan Liu [4], Wenbing Yuan [1], Chengrong Nie [2] and Hailong Wang [1]

1 School of Environmental and Chemical Engineering, Foshan University, Foshan 528000, China
2 School of Food Science and Technology, Foshan University, Foshan 528000, China
3 Foshan Engineering and Technology Research Center for Contaminated Soil Remediation, School of Environmental and Chemical Engineering, Foshan University, Foshan 528000, China
4 School of Environmental Science and Engineering, Key Laboratory of Water Quality and Conservation in the Pearl River Delta, Ministry of Education, Guangzhou University, Guangzhou 510006, China
* Correspondence: bay.beiyuan@fosu.edu.cn

1. Adsorption Kinetics Model

The pseudo-first-order model (Equation S1) and the pseudo-second-order (Equation S2) and the intra-particle diffusion model (Equation S3) were used to fit the sorption kinetic data [50].

$$\text{pseudo-first-order model: } Q_t = Q_e - e^{(\ln(Q_e) - K_1 * t)} \tag{S1}$$

$$\text{pseudo-second-order model: } Q_t = \frac{K_2 * {Q_e}^2 * t}{1 + K_2 * Q_e * t} \tag{S2}$$

$$\text{Intra-particle diffusion: } Q_t = K_3 t^{0.5} + C \tag{S3}$$

Where q_t (mg/g) was the amounts of heavy metals adsorbed at equilibrium and at time t (h), respectively. The K_1 (1/h), K_2 (g/(mg·h)), and K_3 ((mg/g)/$h^{1/2}$) were the pseudo-first-order rate constant, the pseudo-second-order rate constant, and intra-particle diffusion constant, respectively. The C (mg/g) was a constant which reflects the significance of the boundary layer or external mass transfer effect.

2. Adsorption Isotherm Model

The Langmuir model (Eq.4), Freundlich model (Eq.5), and Langmuir Freundlich model (Eq.6) were used to study the sorption isotherm data [51,52].

$$\text{Langmuir model: } Q_e = \frac{K_l * Q_m * C_e}{1 + K_l * C_e} \quad \text{(Eq.4)}$$

$$\text{Freundlich model: } Q_e = K_f * {C_e}^{1/n} \quad \text{(Eq.5)}$$

$$\text{Langmuir-Freundlich model: } Q_e = \frac{K_i * Q_m * {C_e}^{n}}{1 + K_i * C_e} \quad \text{(Eq.6)}$$

Where q_e (mg/g) was the amount of heavy metals adsorbed at equilibrium, q_m (mg/g) was the maximum adsorption capacity of the adsorbent. C_e (mg/L) was the heavy metal concentration of the solution at equilibrium. K_l (mg/L) was the Langmuir constant, K_f was the Freundlich adsorption coefficient, K_i was the Langmuir-Freundlich adsorption coefficient, and n was the adsorption intensity.

Table S1 Dissolved Fe concentration in the P adsorption by CSBC, GBC, and ZBC at pH 1-4. (Initial P concentration=15 mg L^{-1}, solid-to-solution ration=1:200 g mL^{-1}, T=4h, 0.1 M $NaNO_3$, 30 rpm in an end-over-end rotator)

**Biochar**	**Fe (mg L^{-1})**			
	**pH=1**	**pH=2**	**pH=3**	**pH=4**
CSBC	49.22±2.12	7.45±0.73	1.32±0.84	NA
GBC	593.75±25.80	NA	NA	NA
ZBC	30.84±1.54	4.58±0.09	NA	NA

NA: Below the detection value of ICP-OES

Table S2 Maximum adsorption capacity of phosphate by different adsorbents.

Type of adsorbent	Adsorbent	Q_m (mg g^{-1})	Reference
Pristine biochar	Biochar derived from iron−rich sludge	2.643[a]	[53]
	Biochar derived from peanut shell	6.79[a]	[54]
Fe-modified biochar	Iron-impregnated biochars derived from wood chip	3.201[a]	[55]
	Nano-α-Fe_2O_3/Fe_3O_4 supported biochar derived from wood	2.81[a]	[22]
	Magnetic biochar derived from water hyacinth	5.07[b]	[56]
	CSBC	2.88[b]	This study
	GBC	12.33[b]	This study
	ZBC	19.66[b]	This study
Other modified biochar	CO_2 activated biochar derived from Thalia dealbata	4.96[a]	[57]
	Coal gangue modified biochar derived from oilseed rape straw	7.9[a]	[58]
	Mg/Al layered double hydroxide functionalized biochar derived from sugarcane leaves	81.83[a]	[35]
	Magnesium oxide decorated magnetic biochar derived from sugarcane harvest residue	121.25[a]	[43]
	Calcium-alginate beads decorated biochar derived from Laminaria japonica	157.7[b]	[59]
Other adsorbents	Red mud	0.8[b]	[60]
	Magnetic Fe–Zr binary oxide	13.65[a]	[61]
	Magnetic iron oxide nanoparticles	5.03[a]	[62]
	Lanthanum doped vesuvianite	6.7[a]	[63]

Notes: [a] calculated by Langmuir model; [b] calculated by Langmuir-Freundlich model

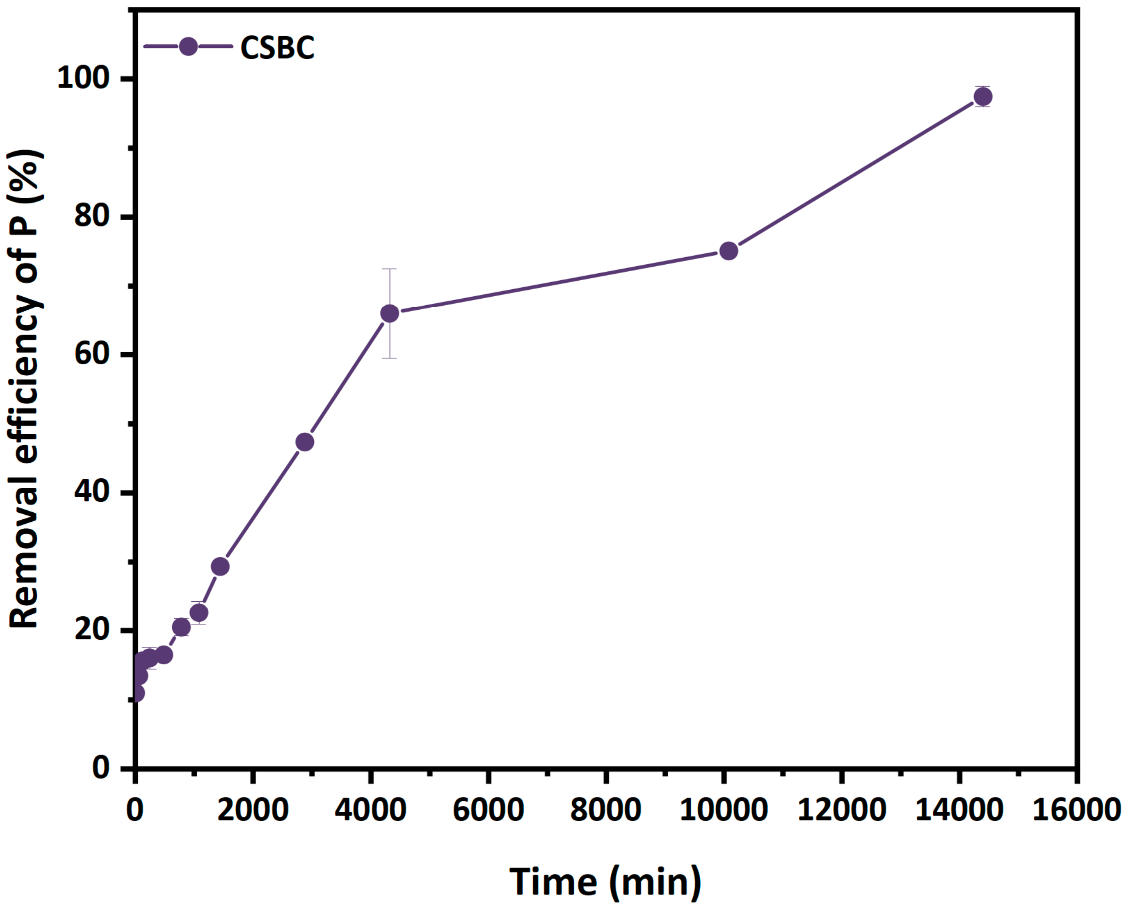

Figure S1 Adsorption kinetics studies of P by CSBC for 10 days

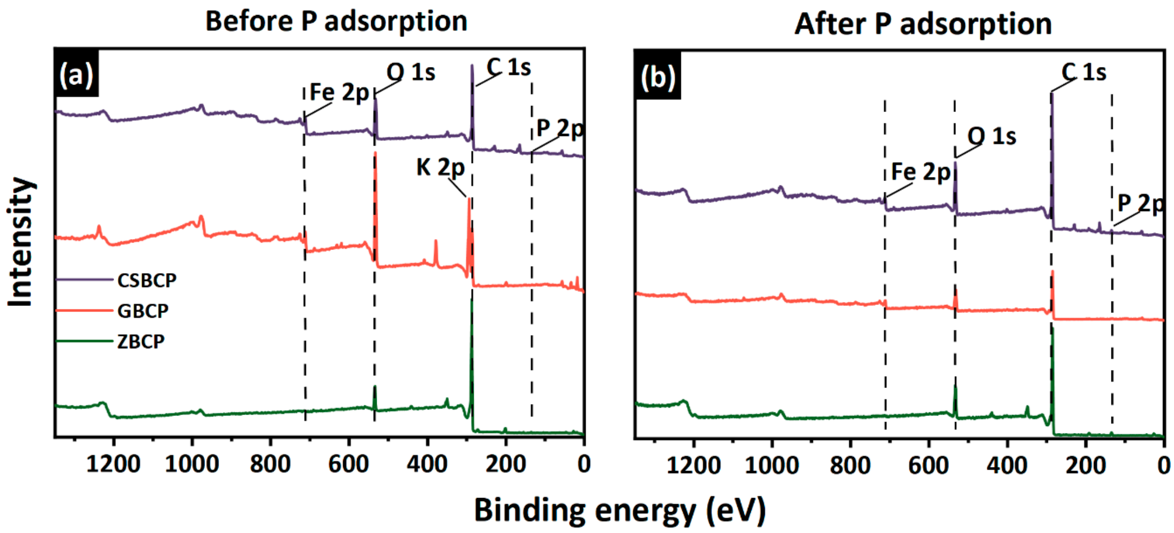

Figure S2 XPS full-scan spectra of Fe-modified biochars before (a) and after P adsorption (b)
